# The Relationship of Personality, Emotional Intelligence, and Aggressiveness in Students: A Study Using the Big Five Personality Questionnaire for Children and Adults (BFQ-NA)

**DOI:** 10.3390/ejihpe11010001

**Published:** 2020-12-25

**Authors:** Jose Luis Antoñanzas

**Affiliations:** Facultad de Educación, Universidad de Zaragoza, 50009 Zaragoza, Spain; jlantona@unizar.es

**Keywords:** personality, emotional intelligence, aggressiveness, youths

## Abstract

An analysis of secondary students’ personality traits, along with a description of their emotional intelligence levels and their anger control, could be decisive when educating students to prevent anti-social behavior in academia. Very few studies on personality, emotional intelligence, and aggressive conduct exist in Spain. Some of the studies that do exist, however, only explore the relationship between emotional intelligence, personality, and prosocial behavior in secondary education students. Likewise, there are few studies focusing on personality and aggression control. In this study, using the Big Five personality models as predictors of aggressiveness in subjects and of emotional intelligence, we sought to contribute to the improvement of the education of students on aggressive behavior in education centers. To do this, we conducted a study using the Big Five Personality Questionnaire (BFQ) for Children and Adults (BFQ-NA), the Trait Meta-Mood Scale (TMMS-24) emotional intelligence test, and the State–Trait Anger Expression Inventory (STAXI) anger management test. Our main objective was to analyze the relationship of the BFQ with the variables of emotional intelligence and aggressiveness. This was achieved using a range of bivariate correlation and multiple regression tests. The results showed the correlation and predictive value of emotional intelligence and aggression in the Big Five model of personality. This study coincides with other research linking Big Five questionnaires with emotional intelligence and aggression.

## 1. Introduction

In the 1980s, the so-called Big Five model introduced the notion, in an empirical manner, that all personality traits could be explained by five dimensions and/or factors. It did this by grouping the behavior and conduct of subjects using a lexical approach, and thus allowed the prediction of psychopathology, juvenile delinquency, school and work performance, normality, risk factors linked to physical health and longevity, etc. [1,2,3]. 

One such example is the personality model of McCrae and Costa [3]. This model appeared using analytical-type, lexical, and temperament factors [4].

Among the questionnaires in the so-called “Five-Factor Model”, apart from the previously cited NEO-PI-R by McCrae and Costa [3]_,_ other instruments exist: *the Zuckerman–Kuhlman Personality Questionnaire* (ZKPQ), *the Big Five Questionnaire* (BFQ) by Caprara, Barbaranelli, Borgogni, and Perugini, and *the Five-Factor Personality Inventory* (5F/PI) by Salgad.

*The Big Five Questionnaire* (BFQ) is one of the so-called self-reporting types and is based mainly on the NEO-PI and NEO-PI-R questionnaires, but it intends to surpass some matters that were neither clarified nor solved by both these questionnaires; e.g., assigning facets to personal dimensions. As pointed out by Pedrero [5], the BFQ comes across as being more parsimonious than its predecessors, better matches the main five-factor theoretical principles, and measures a subject’s tendency to distort data and to offer a “distorted” image of oneself, as other personality questionnaires do (EPQ-R, 16 FP, etc.). Another of its characteristics is that it is validated for the Spanish population and offers T-scores in a results table.

In BFQ questionnaires, we find the Big Five Personality Questionnaire (BFQ) for Children and Adults (BFQ-NA) model, which addresses children and adolescents. It is important to remember that different opinions have been expressed on whether adult personality tests are suitable for earlier ages. A general consensus appears to have been reached about the five main dimensions representing stable personality traits in not only adults, but also in children and early adolescents, [6,7,8]. This questionnaire, as pointed out by Ortiz et al. [9], is derived from the adult questionnaire and is composed of five scales: extraversion—introversion or energy; agreeableness—hostility or pleasantness/cordiality; conscientiousness; neuroticism—emotional stability; and intellect or openness to experience.

### 1.1. Adolescence: Personality, Emotional Intelligence, and Aggressiveness

A fundamental stage in human development is doubtlessly adolescence. According to Erikson [10], a search for personal identity takes place in this life stage, which entails difficulties and disagreements with both family and peers. During this process, the self-concept and self-esteem terms become very important; i.e., the image that adolescents have of themselves and how they think that others see them (cognitive aspects [11,12],along with the evaluation aspects (affective) that the subject makes of his/her image, which correspond to the emotional part: self-esteem. It can be stated that self-concept comprises physical, academic, personal, and social components [13,14]), which give way to differentiation and individualization in relation to others. All of this marks a style of thinking and behavior that leads to being self-assertive in society.

Such differentiation is typical of this life stage, and becoming more independent from the family and personal levels will shape personality in all its cognitive, social, and behavioral aspects [15]. Authors like Martínez-Otero [16] state that this stage of uncertainty about the future and the insecurities that adolescents experience, which are connected with this permanent process of searching for and redefining their identity, can even decrease their mental health.

With all these difficulties and circumstances, in this development stage, to the already known controversies with the family, we can add another series of factors (food, health, etc.) that lead youths to have serious problems as they mature as human beings. Of all these problems, one particularly stands out: youth violence. Adolescents’ aggressive conduct has systematically increased in our society [17,18,19]. This is the reason for why today’s studies of adolescence examine aggressiveness in youths in academic, social, and family settings. Some papers have related aggressiveness in adolescents with personality, and those by Carraco and Del Barrio [20] particularly stand out in this respect. These authors have demonstrated that many factors exist in aggressiveness that can be divided into two large groups: social factors, where solutions are of the political kind, and personal factors, where interventions are mainly of the psychological kind. These authors start with the notion that if the personality factors that predict aggressive conduct can be isolated, a relevant step toward preventing such conduct will have been taken. Like the above-cited authors, in our study, we began with the Big Five Personality Model, which is similar to the Three-Factor model by Eysenck [21] and maintains that aggressiveness correlates with different personality factors. Indeed, neuroticism comes across as a factor that multiplies the risk of aggressiveness due to the lack of control and empowered impulsiveness. Apart from personality, another series of factors can be found that influence the appearance of aggressive conduct.

On the one hand, the concept of emotional intelligence (EI) is very broad, and many definitions have been used in the past. In our study, we used the definition provided by Salovey and Mayer [22]: EI can be defined as a subject’s ability to regulate his or her own and other people’s emotions and/or feelings, and that serves to guide both our thinking and our actions. The way in which EI is measured is also an important consideration. Two main schools of thought exist: one that defines EI as an emotion-related skill, similar to cognitive abilities [23,24] and another one defining EI as a set of emotion-related traits more akin to personality [25]. The latter [26] is evaluated with an instrument comprising short verbal statements, which is called the “Index of perceived or self-reported emotional intelligence”. Within these questionnaires on perceived EI are the Trait Meta-Mood Scale (TMMS; [23]—adapted to Spanish by Fernández-Berrocal et al.[27]—which is the scale used in this study, as well as Schutte’s lE scale [28].

Emotional intelligence (EI) has been systematically and thoroughly studied in the last few decades in relation to youths. It is worth mentioning the studies by Berrocal and Aranda [29], who reviewed works in academic contexts, that by Buenrostro-Guerrero et al. [30] about the relation between EI and academic performance, that by Rodríguez [31] on intervention programs for EI in youths with Down’s syndrome, or that by Martín and Gonzalez [32] about youths’ physical activity with EI programs. Other research works can be found about the use of mobile phones, youths with autism spectrum disorder, and/or parental styles. However, fewer studies refer to the relation with aggressiveness. However, the study by Palomera, Salguero, and Ruiz-Aranda [33], which was conducted with secondary education students, stands out, as its results show how the skill of perceiving emotions is a stable predictor of less clinical and emotional maladjustment and of greater personal adjustment. We particularly highlight the work by Saura et al. [34], who analyzed aggressive behavior in adolescents in relation to EI. Their results indicated that adolescents with high scores in aggressive physical and verbal behavior, hostility, and anger obtained significantly lower scores for the EI trait than their peers with low scores for aggressive physical and verbal behavior, hostility, and anger. Other studies, like that by Garaigordobil and Oñederra [35], have demonstrated that subjects who had been victims of bullying and youths who scored high in antisocial conduct obtained low EI scores.

All these studies relate aggressiveness with EI, while others have also related personality with EI. Authors like Ciarrochi, Chan, and Caputi [36] investigated EI in relation to a series of criterion variables. Their study verified how EI related positively with some personality variables like empathy, self-esteem, extraversion, and openness to feelings. The trait models considered EI to be a series of stable personality traits, socioemotional competence, motivational aspects, and different cognitive skills [37]. In Spain, this has been the most successful working model in organizations until quite recently, due mainly to the impact of Goleman’s book [38,39]

### 1.2. Objective

The main objective of this study was to examine the relationships of the variables of consciousness, agreeableness, emotional instability, extraversion, and openness in the BFQ-NA questionnaire with the variables of perception, clarity, and recovery in the TMMS-24 and with the variables of the control and expression of anger in the State–Trait Anger Expression Inventory (STAXI) to see the BFQ-NA’s power in predicting emotional intelligence and anger in young people.

The variables of age and gender, which are two very important variables in adolescence, were also studied as predictors of personality with the Big Five questionnaire.

## 2. Method

Our sample of participants included 190 students in the 15–18 age group with an average age of 15.8 years (SD = 1.0), of whom 56.8% were males and 43.2% were females.

Basic descriptive methods were followed for the descriptive statistical analysis of the sample. The number of cases in each category and the corresponding percentages were obtained for the qualitative variables, while the minimum and maximum values, means, and standard deviations were obtained for the quantitative variables.

The correlation between variables was studied using Pearson’s correlation coefficient. To determine the influence that demographic variables, the Trait Meta-Mood Scale (TMMS-24), and the State–Trait Anger Expression Inventory (STAXI) had on the BFQ dimensions, hierarchical multiple regression models were used that included as predictor variables the demographic variables of gender and age in Step 1, the TMMS questionnaire dimensions in Step 2, and the STAXI questionnaire dimensions in Step 3. The statistical analysis was done with the SPSS 22.0 program for Windows. Differences were considered significant if *p* < 0.05.

### Procedure

The students who answered the questionnaire were in year 1 of Public High School Education. They received specific instructions as to how to answer the questionnaires from the psychologists in charge of organizing the tests. The tests lasted 90 min. The results were entered into a database. The participation rate was very high (96%) and there were hardly any questionnaires that were not filled in.

## 3. Instruments

The BFQ-NA for personality and the TMMS-24 were used to measure EI, while the STAXI was employed to evaluate aggressiveness.

The BFQ-NA [40] is the model that has been adapted for the Big Five personality traits of children and adolescents. This questionnaire comprises 65 items, are which scored on a scale of from one to five. This questionnaire can be completed by both the child and his/her parents/teachers. It can be used individually or in groups, and its estimated time is roughly 30 min. The results of the study suggest good consistency in all its subscales, with values ranging from 0.5 to 0.87. The degree of reliability found guarantees the correlation between the items evaluated and shows the reliability of the data obtained. The five evaluated dimensions are: awareness (autonomy, orderliness, precision, and compliance with rules and commitments), kindness (concern for and sensitivity to others and their needs), emotional instability (feelings of anxiety, depression, discontent, or anger), extraversion (creativity, enthusiasm, assertiveness, and self-confidence), and openness (cultural interests, creativity, and interest in other people and cultures) [41,42]. As regards the validity of the questionnaire, the internal consistency analysis conducted by Martinez et al. [40] resulted in the subscales of consciousness (α = 0.87), kindness (α = 0.82), emotional instability (α = 0.83), extraversion (α = 0.75), and openness (α = 0.72). The internal consistency analysis for the total scale (n = 65) gave a Cronbach’s alpha value of 0.86, which shows the adequate reliability of the items that make up the scale.

The TMMS-24 is a reduced version of the TMMS-48 by the research group of Fernández-Berrocal, Extremera, and Ramos [26]. It comprises the three original scale dimensions: attention, clarity, and repair; attention to feelings is the ability to identify emotions in oneself and in others; clarity in feelings is the ability to understand emotions in oneself and others; regulation, or emotional repair, is the ability to manage emotions. This questionnaire is composed of 24 items and is answered on a five-point scale. It contains no wrong or right answers, and pays attention only to each individual’s preferences. One very important matter when individuals answer the questionnaire is that they do so quickly and honestly. The internal consistencies reported by the authors for each dimension were attention (α = 0.90), clarity (α = 0.90), and repair (α = 0.86), which indicated internal consistency (α by Cronbach), as well as adequate test–retest reliability.

The STAXI-2 is a questionnaire that describes states of anger and their effects on mental and physical health. It has two basic objectives: first, to determine the components of anger to make an accurate evaluation of normal and abnormal personality; second, to provide an instrument that measures the contributions of different components of anger to the development of certain health problems. It analyzes several components of anger—experience, expression, and control—and its facets, like state and trait. With 49 elements arranged on six scales and five subscales, it allows an index of each scale and subscale, as well as the general test index to be obtained. The analysis of internal consistency by means of Cronbach’s alpha coefficient reached values showing adequate reliability for the different STAXI-2 factors between 0.67 and 0.86 [43].

## 4. Results

The results confirmed a certain correlation between the variables of personality (BFQ-NA), of emotional intelligence (TMSS-24), and of anger or aggression control (STAXI). We found that the clarity and understanding feelings dimensions correlated positively with both these variables in the BFQ-NA. Indeed, the clarity and understanding feelings dimensions correlated with the conscientiousness (0.21 **), openness (0.22 **), and extraversion (0.29 ***) dimensions (see Table 1). In turn, the repairing feelings dimension correlated with extraversion (0.35 ***) and agreeableness (0.29 ***) (Table 1). The same occurred with the STAXI-2 questionnaire, but to a lesser extent, as the STAXI dimension of internal control correlated with the dimensions of conscientiousness (0.21 **), openness (0.20 **), extraversion (0.15 *), and agreeableness (0.29 ***). Moreover, the instability dimension correlated with the anger trait (0.28 **) and with external expression (0.31 ***).

To determine the influence that the TMMS and STAXI demographic variables had on the BFQ dimensions, hierarchical multiple regression models were used, which included the demographic variables of gender and age as predictor variables in Step 1, the TMMS dimensions in Step 2, and the STAXI questionnaire dimensions in the third and final step.

Table 2 provides the results of the model run on the BFQ conscientiousness dimension. As we can see, the demographic variables had no significant effect on conscientiousness (Step 1). When the TMMS (Step 2) dimensions were introduced into the model, the resulting model explained 5.8%, and these dimensions significantly increased, with 5.5% of explained variance, where the clarity dimension was significantly associated with conscientiousness. The complete model (Step 3), to which the STAXI dimensions had been added, explained 14.0% of variance, while the STAXI dimensions explained an additional 8.5% of variance. This increase was statistically significant. In this last model, the demographic variables had no significant effects on conscientiousness, while clarity and internal control had direct and significant effects, such that the greater the clarity and internal control were, the higher the level of conscientiousness was: clarity (B = 0.24, *p* = 0.006) and internal control (B = 0.54, *p* = 0.042); clarity proved to be the best predictor of the conscientiousness score *(r* = 0.21).

Table 3 offers the results of the model run on the BFQ openness dimension. As we can see, the demographic variables had no significant effects on openness (Step 1). When the TMMS dimensions were introduced into the model (Step 2), the resulting model explained 8.0% by significantly increasing these dimensions, with6.1% of variance, where the variables of age and clarity were significantly associated with openness. When the STAXI dimensions were added to the model (Step 3), the explained variance of 7.4% significantly increased to 13.5%. In this model, age had an indirect and significant effect (*p* = 0.038) on openness in such a way that for each year that age increased, the openness score dropped by 1.09 points, whereas clarity and internal control had a direct and significant effect in such a way that the openness score was associated with high levels for these two dimensions—clarity (B = 0.24, *p* = 0.006) and internal control (B = 0.74, *p* = 0.006)—and both were the best predictors of the openness score *(r* = 0.20 in both cases).

The model of the extraversion dimension is found in Table 4. As we can see, the demographic variables had no significant effect on extraversion (Step 1). When the TMMS dimensions (Step 2) were introduced into the model, the resulting model explained 16.2% by significantly increasing these dimensions, with 15.5% of explained variance, where the clarity and repair dimensions were seen to have significant effects on extraversion. When the STAXI dimensions were added to the model (Step 3), the 3.7% explained variance significantly increased and rose to 19.2%. In this model, clarity, repair, and external expression had a direct and significant effect, as the extraversion score was associated with high levels for these dimensions: clarity (B = 0.21, *p* = 0.028), repair (B = 0.36, *r* = 0.26).

The model of the agreeableness dimension is shown in Table 5. As we can see, the age variable had a significant and direct effect on agreeableness (Step 1). When the TMMS dimensions were introduced into the model (Step 2), the resulting model explained 14.0% by significantly increasing these dimensions, with 9.6% of explained variance, where age and the repair dimension had significant effects on agreeableness. When the STAXI dimensions were added to the model (Step 3), explained variance significantly increased by 11.6% and rose to 21.2%. In this model, age had a direct and significant effect (*p* = 0.006) on agreeableness in such a way that for each year that age increased, the openness score increased by 1.54 points, whereas clarity and repair had a direct significant effect in such a way that the agreeableness score was associated with high levels of these dimensions: clarity (B = 0.18, *p* = 0.005) and repair (B = 0.22, *p* = 0.025). Moreover, the anger trait had an indirect and significant effect in such a way that the agreeableness score was associated with low levels of this dimension (B = −0.55, *p* = 0.007). According to the partial correlation coefficients, age and the anger trait were the most relevant variables when predicting the extraversion score *(r* = 0.20 in both cases), followed by repair (*r* = 0.17) and clarity (*r* = 0.15).

Table 6 shows the model of instability. As we can see, the age variable had an indirect and significant effect on instability (Step 1). When the TMMS dimensions were introduced into the model (Step 2), the resulting model explained 6.6%, and these dimensions significantly increased with 3.9% of explained variance. The repair dimension had significant effects on instability, but age was no longer significant. When the STAXI dimensions were added to the model (Step 3), the explained variance of 19.0% significantly increased and became 22.9%. In this model, external expression had a direct and significant effect in such a way that the instability score was associated with high external expression levels (B = 0.92, *p* = 0.019), whereas external control had an indirect and significant effect in such a way that the instability score was associated with low levels of this dimension (B = −1.28, *p* < 0.001). None of the other variables had a significant effect on instability. According to the partial correlation coefficients, the external control variable was the most relevant in the prediction of the instability score *(r* = −0.26).

## 5. Discussion and Conclusions

As our results show, the BFQ was correlated with EI and with anger, just as Carrasco and Del Barrio [20] demonstrated in their study with adolescents, where the BFQ was a good predictor of aggressiveness. Our study sought to predict BFQ factors with the questionnaires of the TMSS-24, which measures EI, and the STAXI, which measures the anger state–trait. The TMSS-24 was first introduced, followed by STAXI, to see the predictive values of the various factors. With the conscientiousness and openness dimensions, aggressiveness was explained by the clarity (TMSS) and internal control (STAXI) dimensions in such a way that the greater values of clarity and internal control were, the greater the values of conscientiousness and openness became. Understanding feelings, along with greater internal control, allowed the subjects to have better knowledge of their states and, in turn, to make decisions that better matched reality. Along the same lines, a study conducted by Ingles et al. [44] demonstrated that low levels of EI were correlated with less anger control and, therefore, with higher levels of aggressiveness; that is, not understanding emotions and internal controls means a worse state of aggressiveness. Other studies, like those by Palomera, Salguero, and Ruiz-Aranda, [35], have also shown how emotional skills, clarity, and understanding emotions conferred adolescents with better social adjustment, as they were able to maintain better social relationships with both peers and family. These same results remained even after controlling for variables like age and gender, as well as for important personality dimensions. These results also coincide with those of the present study. The extraversion and agreeableness dimensions were explained by the variables of clarity and repair of emotions and by external expression, but with a heavier specific weight for repairing and/or regulating emotions, such that the greater the emotional repair score was, the higher the extraversion score became.

It is worth stressing other studies, like those by Martorel, Gonzalez, Rasal, and Estelles [45], in which youths with low EI, which is understood as a trait, found it more difficult to relate with others precisely because they lacked emotional self-control and empathy.

We conclude that the questionnaires of the TMSS-24 and STAXI are good predictors of the BFQ-NA personality dimensions. Although this study has a series of limitations—e.g., its sample—the data that it obtained are significant, as very few studies have related EI with aggressiveness and personality in adolescents. Continuing to investigate these variables is recommended because, as we have seen, they are very important for controlling anti-social behavior in education centers and, therefore, in devising intervention programs.

## Figures and Tables

**Table 1:** Means, standard deviations, and correlations of the Big Five Personality Questionnaire (BFQ), Trait Meta-Mood Scale (TMMS-24), and State–Trait Anger Expression Inventory (STAXI) dimensions.

Variable Mean(SD)	1	2	3	4	5	6	7	8	9	10	11	12	13
1. Conscientiousness 46.21 (7.10)	1												
2. Openness 47.91 (7.11)	0.73 ***	1											
3. Extraversion 47.27 (8.14)	0.38 ***	0.28 ***	1										
4. Agreeableness 50.25 (7.98)	0.56 ***	0.36 ***	0.50 ***	1									
5. Instability 51.95 (9.99)	−0.04	−0.01	−0.02	−0.22 **	1								
6. Attention 25.17 (6.60)	0.13	0.13	0.07	0.04	0.09	1							
7. Clarity 24.83 (6.24)	0.21 **	0.22 **	0.29 ***	0.21 **	−0.09	0.14	1						
8. Repair 27.26 (6.30)	0.12	0.11	0.35 ***	0.29 ***	−0.18 *	0.05	0.29 **	1					
9. Anger State 8.58 (1.83)	−0.12	−0.02	−0.13	−0.05	0.14	−0.02	−0.11	−0.23 **	1				
10. Anger Trait 14.24 (3.28)	−0.19 **	−0.1	0.01	−0.20 **	0.28 ***	0.09	−0.03	−0.11	0.38 ***	1			
11. External expression 8.13 (1.96)	−0.13	−0.08	0.12	−0.06	0.31 ***	0.10	0.10	−0.09	0.19 **	0.46 ***	1		
12. Internal expression 7.22 (1.78)	0.10	−0.02	−0.13	0.04	0.03	0.21 **	−0.16 *	−0.04	0.13	0.16 *	0.02	1	
13. External control 8.60 (2.23)	0.14	0.06	0.08	0.25 **	−0.38 **	0.03	0.13	0.30 ***	−0.21 **	−0.29 ***	−0.28 ***	0.09	1
14. Internal control 8.70 (2. 31)	0.21 **	0.20 **	0.15 *	0.29 ***	−0.19 *	−0.08	0.10	0.43 ***	−0.22**	−0.26 ***	−0.21 **	0.03	0.49 ***

SE: standard error. * *p* < 0.05; ** *p* < 0.01; *** *p* < 0.001.

**Table 2:** The multiple regression analysis for conscientiousness.

Predictor	Step 1			Step 2			Step 3		
	B(SE)	*t*	Partial *r*	B(SE)	*t*	Partial *r*	B(SE)	*t*	Partial *r*
Gender	0.67 (1.06)	0.63	0.05	0.49 (1.07)	0.46	0.03	0.24 (1.04)	0.23	0.02
Age	−0.15 (0.53)	−0.28	−0.02	−0.23 (0.52)	−0.44	−0.03	−0.33 (0.52)	−0.65	−0.05
Attention				0.10 (0.08)	1.18	0.09	0.11 (0.08)	1.31	0.10
Clarity				0.20 (0.09)	2.32 *	0.17	0.24 (0.09)	2.80 **	0.21
Repair				0.08 (0.09)	0.91	0.07	−0.03 (0.09)	−0.35	−0.03
Anger state							−0.09 (0.30)	−0.30	−0.02
Anger trait							−0.32 (0.19)	−1.69	−0.13
External expression							−0.25 (0.29)	−0.85	−0.06
Internal expression							0.54 (0.30)	1.80	0.13
External control							−0.13 (0.27)	−0.49	−0.04
Internal control							0.54 (0.27)	2.05 *	0.15
**R2 (%)**	0.3			5.8			14.0		
**^∆R2^ (%)**				5.5			8.5		
**Model**	F (2.189) = 0.27			F (5.189) = 2.25 *			F (11.189) = 2.63 **		

SE: standard error. * *p* < 0.05; ** *p* < 0.01; *** *p* < 0.001.

**Table 3:** The multiple regression analysis for openness.

Predictor	Step 1			Step 2			Step 3		
	B(SE)	*t*	Partial *r*	B(SE)	*t*	Partial *r*	B(SE)	*t*	Partial *r*
Gender	0.02 (1.05)	0.02	0.00	−0.22 (1.06)	−0.20	−0.02	−0.44 (1.05)	−0.42	−0.03
Age	−0.99 (0.53)	−1.86	−0.14	−1.06 (0.52)	−2.05 *	−0.15	−1.09 (0.52)	−2.09 *	−0.16
Attention				0.11 (0.08)	1.42	0.10	0.16 (0.08)	1.94	0.14
Clarity				0.22 (0.09)	2.53 *	0.18	0.24 (0.09)	2.76 **	0.20
Repair				0.06 (0.08)	0.76	0.06	−0.03 (0.09)	−0.38	−0.03
Anger state							0.24 (0.30)	0.79	0.06
Anger trait							−0.10 (0.19)	−0.54	−0.04
External expression							−0.32 (0.29)	−1.10	−0.08
Internal expression							−0.01 (0.30)	−0.04	0.00
External control							−0.26 (0.27)	−0.96	−0.07
Internal control							0.74 (0.27)	2.77 **	0.20
**R2 (%)**	1.9			8.0			13.5		
**^∆R2^ (%)**				6.1			7.4		
**Model**	F (2.189) = 1.78			F (5.189) = 3.22 **			F (11.189) = 2.53 **		

SE: standard error. * *p* < 0.05; ** *p* < 0.01; *** *p* < 0.001.

**Table 4:** The multiple regression analysis for extraversion.

Predictor	Step 1			Step 2			Step 3		
	B(SE)	*t*	Partial *r*	B(SE)	*t*	Partial *r*	B(SE)	*t*	Partial *r*
Gender	0.26 (1.21)	0.22	0.02	0.51 (1.16)	0.44	0.03	0.71 (1.16)	0.61	0.05
Age	0.69 (0.61)	1.12	0.08	0.46 (0.57)	0.81	0.06	0.53 (0.58)	0.91	0.07
Attention				0.03 (0.09)	0.35	0.03	0.04 (0.09)	0.49	0.04
Clarity				0.26 (0.09)	2.81 **	0.20	0.21 (0.10)	2.22 *	0.16
Repair				0.37 (0.09)	4.03 ***	0.29	0.36 (0.10)	3.55 ***	0.26
Anger state							−0.26 (0.34)	−0.78	−0.06
Anger trait							0.07 (0.21)	0.33	0.03
External expression							0.54 (0.23)	2.38 *	0.12
Internal expression							−0.46 (0.33)	−1.40	−0.10
External control							−0.06 (0.30)	−0.21	−0.02
Internal control							0.15 (0.30)	0.50	0.04
**R2 (%)**	0.7			16.2			19.2		
**^∆R2^ (%)**				15.5			3.7		
**Model**	F (2.189) = 0.63			F (5.189) = 7.13 ***			F (11.189) = 3.84 ***		

SE: standard error. * *p* < 0.05; ** *p* < 0.01; *** *p* < 0.001.

**Table 5:** The multiple regression analysis for agreeableness.

Predictor	Step 1			Step 2			Step 3		
	B(SE)	*t*	Partial *r*	B(SE)	*t*	Partial *r*	B(SE)	*t*	Partial *r*
Gender	0.57 (1.16)	0.49	0.04	0.85 (1.15)	0.74	0.05	0.43 (1.12)	0.38	0.03
Age	1.73 (0.59)	2.94 **	0.21	1.55 (0.56)	2.75 **	0.20	1.54 (0.56)	2.76 **	0.20
Attention				0.01 (0.09)	−0.01	0.01	0.03 (0.09)	0.29	0.02
Clarity				0.18 (0.09)	1.92	0.14	0.18 (0.09)	1.97 *	0.15
Repair				0.31 (0.09)	3.37 **	0.24	0.22 (0.10)	2.27 *	0.17
Anger State							0.56 (0.33)	1.72	0.13
Anger Trait							−0.55 (0.20)	−2.75 **	−0.20
External expression							0.31 (0.32)	0.98	0.07
Internal expression							0.28 (0.32)	0.87	0.07
External control							0.21 (0.29)	0.74	0.06
Internal control							0.46 (0.29)	1.60	0.12
**R2 (%)**	4.4			14.0			21.2		
**^∆R2^ (%)**				9.6			11.6		
**Model**	F (2.189) = 4.33 *			F (5.189) = 5.99 ***			F (11.189) = 4.37 ***		

SE: standard error. * *p* < 0.05; ** *p* < 0.01; *** *p* < 0.001.

**Table 6:** The multiple regression analysis for instability.

Predictor	Step 1			Step 2			Step 3		
	B(SE)	*t*	Partial *r*	B(SE)	*t*	Partial *r*	B(SE)	*t*	Partial *r*
Gender	0.96 (1.47)	0.65	0.05	0.30 (1.50)	0.20	0.02	0.65 (1.39)	0.47	0.04
Age	−1.54 (0.74)	−2.08 *	−0.15	−1.41 (0.73)	−1.93	−0.14	−1.07 (0.69)	−1.54	−0.12
Attention				0.15 (0.11)	1.29	0.10	0.11 (0.11)	0.98	0.07
Clarity				−0.09 (0.12)	−0.78	−0.06	−0.10 (0.12)	−0.90	−0.07
Repair				−0.25 (0.12)	−2.12 *	−0.15	−0.13 (0.12)	−1.03	−0.08
Anger State							−0.14 (0.40)	−0.35	−0.03
Anger Trait							0.40 (0.25)	1.60	0.12
External expression							0.92 (0.39)	2.36 *	0.17
Internal expression							0.04 (0.40)	0.10	0.01
External control							−1.28 (0.36)	−3.57 ***	−0.26
Internal control							0.32 (0.35)	0.91	0.07
R2 (%)	2.7			6.6			22.9		
^∆R2^ (%)				3.9			19.0		
Model	F (2.189) = 2.64			F (5.189) = 2.60 *			F (11,189) = 4,80 ***		

SE: standard error. * *p* < 0.05; ** *p* < 0.01; *** *p* < 0.001.

## Data Availability

The data presented in this study are available on request from the author.
